# North Atlantic forcing of moisture delivery to Europe throughout the Holocene

**DOI:** 10.1038/srep24745

**Published:** 2016-04-25

**Authors:** Andrew C. Smith, Peter M. Wynn, Philip A. Barker, Melanie J. Leng, Stephen R. Noble, Wlodek Tych

**Affiliations:** 1 Lancaster Environment Centre, Lancaster University, Lancaster, LA1 4YQ, UK; 2 NERC Isotope Geosciences Facility, British Geological Survey, Nottingham, NG12 5GG, UK; 3 Centre for Environmental Geochemistry, University of Nottingham, Nottingham, NG7 2RD, UK.

## Abstract

Century-to-millennial scale fluctuations in precipitation and temperature are an established feature of European Holocene climates. Changes in moisture delivery are driven by complex interactions between ocean moisture sources and atmospheric circulation modes, making it difficult to resolve the drivers behind millennial scale variability in European precipitation. Here, we present two overlapping decadal resolution speleothem oxygen isotope (δ^18^O) records from a cave on the Atlantic coastline of northern Iberia, covering the period 12.1–0 ka. Speleothem δ^18^O reveals nine quasi-cyclical events of relatively wet-to-dry climatic conditions during the Holocene. Dynamic Harmonic Regression modelling indicates that changes in precipitation occurred with a ~1500 year frequency during the late Holocene and at a shorter length during the early Holocene. The timing of these cycles coincides with changes in North Atlantic Ocean conditions, indicating a connectivity between ocean conditions and Holocene moisture delivery. Early Holocene climate is potentially dominated by freshwater outburst events, whilst ~1500 year cycles in the late Holocene are more likely driven by changes internal to the ocean system. This is the first continental record of its type that clearly demonstrates millennial scale connectivity between the pulse of the ocean and precipitation over Europe through the entirety of the Holocene.

Holocene (~11.7–0 ka) climate is characterised by a series of century-to-millennial scale changes which have had varied regional impacts^1^,^2^,^3^, resulting in relatively colder polar regions, more arid tropics and major fluctuations in atmospheric circulation systems^4^,^5^,^6^. Such climatic variability has been attributed to solar cycles^3^,^7^,^8^, changes in North Atlantic Ocean circulation^9^,^10^,^11^,^12^ and variations in atmospheric modes similar to the modern day North Atlantic Oscillation (NAO)^2^,^13^,^14^. Rather than these Holocene events being temporally random, palaeoclimate records have been used to propose millennial scale cycles, the three most widely recognised periodicities being at approximately 2000–2800 years^5^, 1500 years^3^,^6^,^11^,^15^ and 1000 years^2^,^9^. The 1500 year periodicity is most clearly displayed in the North Atlantic region from ocean sediments, which demonstrate millennial scale peaks in the southerly transport of ice rafted debris (IRD)^3^,^16^. Nine such ocean cooling events (Bond events, labelled 0–8) have been identified during the Holocene and were initially believed to be directly related to variations in solar activity^3^. This proved controversial and more recently the ~1500 year period has been attributed to internal oceanic forcing^9^,^10^,^12^ possibly representing a threshold response in North Atlantic Ocean circulation to external solar cycles^17^. Vestiges of these ~1500 year cycles are identifiable in the North Atlantic Ocean^3^,^12^, although many North American and European terrestrial records fail to recognize more than a selection of similarly timed climatic events during the Holocene^6^,^18^ and few, if any, records exhibit cycles through the full Holocene. This discrepancy causes uncertainty about the hemispheric impact of ‘Bond cycles’ on terrestrial climate and how changes in the ocean are transmitted to the climate system.

Here, we add to this debate by reconstructing precipitation delivery to northern Spain during the last 12.1 ka, using a carbonate oxygen isotope (δ^18^O) record derived from speleothems contained within Cueva de Asiul (43° 19′ 0.63″ N, 3° 35′ 28.32″ W; 285 m.a.s.l) situated in the Matienzo depression (Cantabria), N. Spain^19^ (Fig. 1). At this location, approximately 80% of the region’s annual precipitation is derived from an Atlantic source, associated with westerly frontal systems^19^,^20^,^21^,^22^. The monthly isotope composition of modern rainfall (monthly weighted mean) is controlled by a rainfall amount effect (r^2^ = 0.51 p < 0.01), with lower δ^18^O related to higher rainfall totals^19^. At such mid-latitudes, it is variations in the mode of the NAO, defined as the difference in pressure between the Icelandic low and the Azores high^23^, which are the primary control over the relative positioning of westerly wind tracks across Europe^24^,^25^,^26^,^27^,^28^ (Fig. 1), the amount of inter-annual winter precipitation in north western Iberia (r^2^ = 0.6; 1948–2008 AD)^29^ and the δ^18^O of rainfall^21^.

Traditionally, positive NAO (NAO+) conditions force winter storm tracks associated with the Atlantic Westerly Jet (AWJ) to migrate northwards^23^, leading to wetter and warmer winter conditions in north-western Europe and drier conditions in southern Europe, including the Iberian Peninsula^29^,^30^. Under negative NAO (NAO−), storm tracks weaken and shift southwards reversing the pattern^23^. In addition to inter-annual control over European climate, NAO-like conditions are thought to persist over longer centennial timescales, due to a relatively stable positioning of the AWJ during periods of the Holocene^1^,^24^.

The two speleothems used in this study (ASR and ASM) grew just 5 m apart, in the Cueva de Asiul system in a stable cave environment (air temp 13.7 ± 0.5 °C)^19^,^31^ (75 m in length, 40 m rock overburden) and were both actively depositing calcite at the time of removal. The cave’s hydrological system is recharged primarily by winter rainfall (1400 mm/year), resulting in cave drip waters that reflect a bulk winter rainfall δ^18^O composition from the preceding year^19^. The analysis of modern carbonates indicates a minimal level of isotopic disequilibrium within the system^19^,^32^. When combined, the speleothem δ^18^O records provide an almost complete Holocene archive of precipitation amount with a resolution of between 1 and 28 years/ data point (Fig. 2), with the chronology based on 22 U/Th analyses. Our combined speleothem δ^18^O data demonstrate correlation with other palaeorecords of NAO^24^ up to 2.5 ka (see supplementary information) and in total show nine distinctly dry periods during the Holocene. These nine phases coincide with the timing of recorded ocean cooling events^3^, indicating a potential Holocene duration connectivity between oceanic temperature and major changes in modes of atmospheric circulation.

## Results

**Figure 2:**
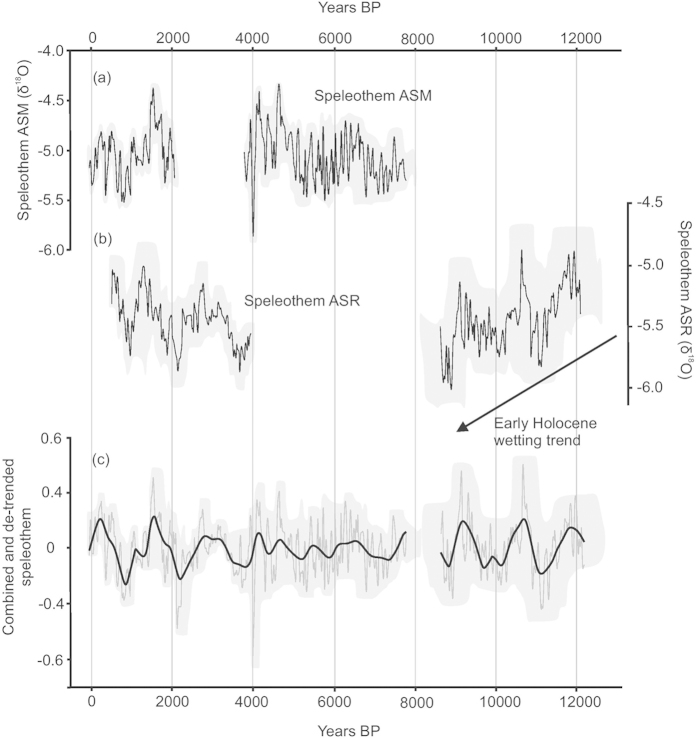
Cueva de Asiul speleothem δ^18^O data. (**a**) Raw speleothem δ^18^O (‰, VPDB) for sample ASM and (**b**) ASR, grey boundaries denote the measurement error (0.1‰) and the variable timing error based on the StalAge model. (**c**) Combined and detrended speleothem data (thin grey line) with the underlying trend of this data (black line) as defined by DHR analysis (see supplementary information), again grey boundaries denote the associated errors.

The relatively large amplitude (1–2‰) peaks in carbonate δ^18^O which occur throughout the Cueva de Asiul data provide timings for reductions in moisture delivery (Fig. 2). Frequency analysis of the δ^18^O data reveals underlying millennial scale cycles in this precipitation delivery record. During the early Holocene, precipitation amounts follow a slightly shorter 1290 year cycle until 6.5 ka, after which point a longer periodicity (1490 year) cycle becomes dominant. The changing cycle amplitudes and periodicities have been estimated using Dynamic Harmonic Regression^33^,^34^ (see supplementary information). This dual period frequency model explains 74% (r^2^ = 0.74) of the variance observed in the remainder of the speleothem record with its residuals not indicating any significant shorter or longer term cycles.

The beginning of the Cueva de Asiul speleothem record coincides with the latter part of the Younger Dryas (Fig. 2) and confirms this as a period of enhanced aridity in northern Iberia^35^, consistent with the northerly migration of the AWJ as previously identified in other Iberian speleothem records^21^. Transition into the early Holocene is marked by cycles in moisture availability, superimposed upon a general wetting trend until 8.8 ka^30^,^35^ (Fig. 2). These cycles are visible even during glacial boundary conditions prior to complete melting of the Laurentide and Scandinavian ice sheets^36^. At 8.6 ± 0.4 ka speleothem ASR stopped growing, associated with drier and colder conditions throughout southern Europe^37^ during the “8.2 ka” event^38^,^39^, which is known to have had an impact on the climate of this region^40^. Growth in speleothem ASM started at 7.7 ka, suggesting wetter climatic conditions returned to N. Spain during the mid-Holocene thermal optimum^41^. The late Holocene is dominated by four arid periods, one of which corresponds to the well-known “4.2 ka” drought^35^,^42^, extending the known spatial influence of this drought to the western fringes of Europe.

The last 2.5 ka of the record broadly agrees with recent high resolution reconstructions of NAO conditions from other European archives^24^, thereby qualifying the role of the NAO in driving the moisture record within these speleothems (see supplementary information). Increases in rainfall associated with NAO- and a southward shift in the AWJ are observed at 2.1–2.2 ka, 1.4 ka and during the Little Ice Age (0.5–0.3 ka)^1^,^24^. Whereas more NAO+ conditions and lower rainfall amounts are recorded between 2.0–1.5 ka, confirming previous work which suggested a shift to NAO+ conditions prior to the onset of the Medieval Climate Anomaly (MCA)^24^. The following 1000 years including the MCA is characterised by fluctuating^24^,^43^ but predominantly wetter conditions in north-western Spain, with distinctly drier episodes suggesting NAO+ at 1.0, 0.85, 0.7 and 0.6 ka^1^,^24^. There is little evidence in our record for a persistently NAO+ during the MCA as found in some other European reconstructions^44^,^45^. This discrepancy is potentially due to the close proximity of this site to the North Atlantic coastline, with wetter conditions found in other MCA proxy records from the Atlantic coastline of NW. Iberia^45^. This discrepancy in MCA proxy records across Iberia requires further investigation, but potentially indicates a slightly more southerly positioning of storm tracks associated with NAO+ during the MCA.

## Discussion

**Figure 3:**
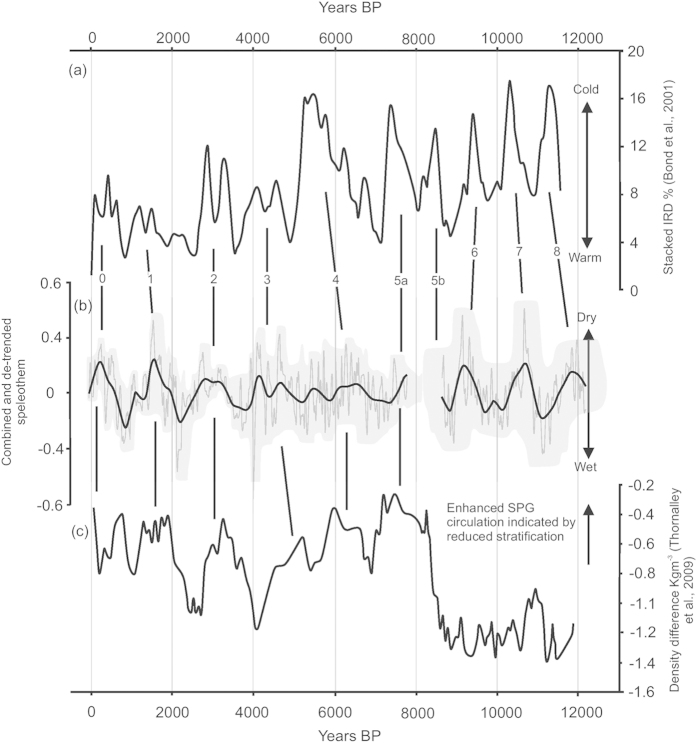
North Atlantic proxy records which highlight millennial scale changes in ocean and atmospheric systems. (**a**) Haematite stained grain (HSG) percentages from four stacked ocean cores^3^ indicating changes in North Atlantic ice rafted debris (IRD) and therefore ocean surface temperature, black vertical lines represent co-variation between this record and (**b**) the combined Cueva de Asiul speleothem δ^18^O record, with grey boundaries denoting the timing error based on the StalAge model, numbers identify Bond events, (**c**) changes in surface and subsurface density difference in the North Atlantic as a proxy for SPG strength and change in fresh water influx^11^. Black lines indicate early Holocene connections between changing ocean circulation strengths and northern Iberian precipitation amount. Cross correlation analysis (see supplementary information) between the Asiul cave archive and the IRD data^3^ are (0–4000 BP, r^2^ = 0.505, standard error = 0.125; 0–7800 BP r^2^ = 0.165, standard error = 0.09; 9000–12,000 BP (t = −390) r^2^ =  0.560, standard error = 0.106) and compared ocean density^11^ data are (0–7800 BP r^2^ = 0.34, standard error = 0.09).

Variations in the Cueva de Asiul speleothem moisture record are shown to occur on a range of temporal scales. Decadal to centennial fluctuations during the last 2.5 ka broadly agree with other European archives of the NAO^24^ (see supplementary information), whereas on a millennial time scale phases of reduced moisture availability are observed with a ~1500 year periodicity throughout the Holocene, co-inciding with North Atlantic Ocean cold periods and increases in North Atlantic IRD^3^ (Fig. 3). This correlation between the atmospheric moisture record from Cueva de Asiul and North Atlantic IRD records (see supplementary information) raises two prominent questions, 1) how are North Atlantic Ocean temperatures and the position of the AWJ connected and 2) which components of the climate system act to propagate a quasi-1500 year cycle in both the ocean and atmospheric records? These two questions are intrinsic to much of the current debate surrounding Holocene climate in the North Atlantic^3^,^30^,^46^,^47^. The correlation between drying in the speleothem record and North Atlantic cold events points to underlying mechanisms of forcing which have operated throughout the Holocene.

During the early Holocene, North Atlantic Ocean conditions were significantly influenced by meltwater outburst events^39^, a direct result of global deglaciation. Outbursts cooled and freshened ocean surface waters, slowing and shifting sites of deep water formation^48^,^49^ and interrupting the northerly transport of warm waters by the thermohaline circulation^39^. Such major changes in ocean boundary conditions would reduce surface evaporation and northward heat transport, potentially resulting in widespread cooling throughout the North Atlantic region. Our speleothem moisture records confirm findings from other records in southern Europe, which suggest significant drying in the Mediterranean^2^ during freshwater outburst events^49^,^50^; the most dramatic of which occurred at 8.2 ka^37^,^39^ and appears to have resulted in a complete stop in speleothem growth (8.6 ± 0.4 ka). The dominance of freshwater outburst events during the early Holocene appears to have been the major control over millennial scale North Atlantic climate perturbations.

At the end of deglaciation the impact of freshwater release into the North Atlantic is reduced, co-inciding with a change in periodicity observed in ocean records^3^,^11^, records of western Mediterranean aridity^2^ and the Cueva de Asiul speleothem archive (Fig. 3). Speleothem δ^18^O identifies ~6.5 ka as a marker for a change in this millennial cycle and the start of a longer period cycle, often quoted at 1600–1800 years^2^ in other European records, but seen as ~1500 years in the Cueva de Asiul speleothems and ocean proxies^3^,^9^,^46^. Disparities between archives are likely a result of age model uncertainties^9^. The emergence of this longer climate cycle in both North Atlantic Ocean circulation^3^,^11^ and regional atmospheric archives^2^,^12^,^51^ points to the development of a millennial scale pacing mechanism or series of feedbacks capable of influencing both oceanic and atmospheric conditions in the absence of freshwater outburst events.

The longer memory effects of the ocean^46^ and its potential to respond to weak solar forcing^17^ could indicate an oceanic source for the millennial scale (~1500 years) climate variability seen during the late Holocene^2^,^11^,^46^. One possible driving mechanism is the strength of the Atlantic Meridional Overturning Circulation (AMOC), which is believed to have switched between a strong and weak state^52^,^53^. These fluctuations in AMOC have been postulated as a control over the development of a North Atlantic temperature dipole similar to the NAO^2^,^52^. Where a weak AMOC causes a deepening of the Icelandic low and an increase in wind intensity over Iceland^52^, resembling NAO+ conditions and enhancing wind driven transport of Atlantic surface waters into the Nordic Sea^2^,^52^. Increased wind stress on the ocean surface can additionally change the strength and/or positioning of ocean circulation systems^30^,^54^,^55^, including the Sub Polar Gyre (SPG)^11^, possibly re-enforcing the climate perturbation and enabling its persistence over longer time scales. When considered in the light of modern conditions, where NAO+ acts to enhance polar outflow and IRD transport through the Fram Strait into the North Atlantic^13^,^55^, a deepening of the Icelandic low in response to weak AMOC could trigger NAO+ like conditions. Enhanced wind stress would in turn cause increases in SPG strength^11^ and North Atlantic IRD outflow^3^,^10^,^13^, as well as causing drier conditions in southern Europe (Fig. 3), including at Cueva de Asiul and in the Mediterranean^2^.

In identifying nine cycles of wetting and drying in northern Iberia during the Holocene, the Cueva de Asiul speleothem data offer a significant contribution to existing terrestrial climate records in Europe. The correlation between these wet and dry events and changes in the North Atlantic Ocean (Fig. 3)^3^,^11^ highlight connectivity between the ocean and atmospheric systems. During the early Holocene the influence of freshwater outburst events appears to dominate the shorter 1290 year climate cycle. The observed change in wet/dry cycle lengths between the early and late Holocene are possibly related to the completion of global deglaciation at ~6.5 ka and a reduction in the influence of freshwater outburst events on North Atlantic Climate. The late Holocene is dominated by a longer (1490 year) cycle, similar to that observed within other European archives^2^,^9^, indicating continued connectivity between the oceanic and atmospheric records. The most likely cause of this millennial scale climate variability originates in the ocean^2^, potentially related to changes in the state of the AMOC which promotes fluctuations in atmospheric circulation in a meridional NAO like pattern causing a repositioning of the AWJ^2^. Once established, NAO+ conditions and a deepening of the Icelandic low potentially promote further changes in ocean circulation due to enhanced wind stress^11^, causing a stabilisation of NAO like conditions over longer time periods. Our findings highlight the complexity of the coupled ocean/ atmosphere system especially during the latter part of the Holocene and show the requirement for further investigation into the driving mechanisms and feedbacks critical to forcing natural climate variability during our most recent interglacial period.

## Methods

The combined chronology is based upon 10 U-Th ages from speleothem ASR and 12 U-Th ages from ASM (uncertainties scale with absolute age, 2σ uncertainty = ±2–20%; see supplementary information). All quoted ages are corrected to calendar years BP (1950) unless stated, from the date of U series analysis (2012). These samples were milled perpendicular to the speleothem’s vertical growth axis; producing 50–100 mg of powder for each sample. Chemical separations for U and Th (class 100 clean lab conditions) and mass spectrometry (Thermo Neptune Plus MC-ICP-MS) were undertaken at the Geochronology and Tracers Facility at the British Geological Survey. Final age models for each speleothem (supplementary information) were constructed using the StalAge Monte-Carlo simulation^56^.

The speleothem δ^18^O profile consists of 1244 isotope measurements from ASR and 880 from ASM, sampled using an automated micro-mill with 0.3 mm diamond encrusted drill bit along the central portion of the growth axis, creating between 50–100 μg of carbonate powder per sample. Stable isotope analysis was then undertaken at the Stable Isotope Facility, British Geological Survey, using an IsoPrime isotope ratio mass spectrometer with Multiprep device; average 2σ uncertainty is 0.07‰. Isotope values are reported relative to the international VPDB standard.

Time series and frequency domain data analysis was performed using Dynamic Harmonic Regression method^23^ and CAPTAIN Toolbox for Matlab^24^.

## Additional Information

**How to cite this article**: Smith, A. C. *et al.* North Atlantic forcing of moisture delivery to Europe throughout the Holocene. *Sci. Rep.* **6**, 24745; doi: 10.1038/srep24745 (2016).

## Supplementary Material

Supplementary Information

## Figures and Tables

**Figure 1:**
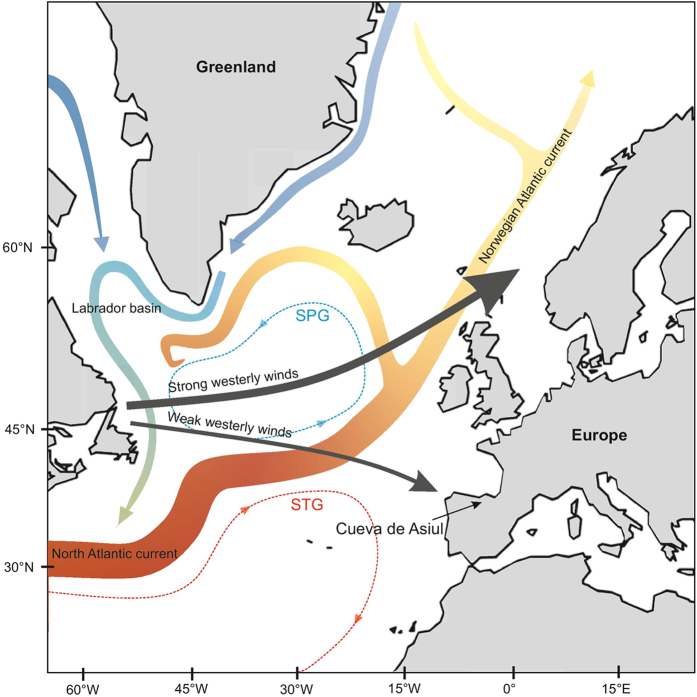
Map of the North Atlantic region. Highlighting the position of Cueva de Asiul and showing the positioning of major thermohaline ocean currents, the sub polar and subtropical gyres and the variable positioning of westerly wind tracks under positive and negative NAO conditions. Image created by ACS in CorelDRAW X6; http://www.coreldraw.com.
