# Supplementary material for: North Atlantic forcing of moisture delivery to Europe throughout the Holocene
Source: Sci Rep. 2016 Apr 25;6:24745. doi: 10.1038/srep24745 (PMC4842961; doi:10.1038/srep24745)
Supplement: Supplementary Information [file srep24745-s1.pdf]

North Atlantic forcing of moisture delivery to Europe throughout the Holocene

Andrew C. Smith[1, 2]*, Peter M. Wynn[1], Philip A. Barker[1], Melanie J. Leng[2, 3], Stephen R. Noble[2] and Wlodek Tych[1]

[1] Lancaster Environment Centre, Lancaster University, Lancaster, LA1 4YQ

[2] NERC Isotope Geosciences Facility, British Geological Survey, Nottingham, NG12 5GG

[3] Centre for Environmental Geochemistry, University of Nottingham, Nottingham, NG7 2RD

* email: andrews@bgs.ac.uk

Supplementary information

Speleothem U-series geochronology

U-series speleothem dating was done at the NERC Isotope Geosciences Laboratory Geochronology and Tracers Facility (NIGL-GTF), all quoted ages are corrected to calendar years BP (1950) from the date of U series analysis (2013). The Cueva de Asiul speleothems were sampled using a New Wave Micromill, with between 50-100 mg of powdered clean carbonate obtained from tracks milled parallel to the growth bands. All geochronology sampling and analytical work was done in a class 100 HEPA filtered clean lab. High purity water (resistivity = 18 MΩ) was prepared using a Milli Q purification system, HCl and HNO_3 were prepared by double sub-boiling distillation in quartz, and high purity (UpA grade, triple Teflon distilled) concentrated ammonia, HF and H_2O_2 were obtained from Romil Ltd.

Sample powders were weighed and placed in Savillex vials, covered in high purity water, and dissolved by drop-wise addition of 15 M HNO_3 to ensure controlled and contained sample dissolutions. Samples were centrifuged and visually checked for complete dissolution and then spiked with a mixed high purity ^{229}Th-^{236}U tracer. The U-Th tracer was calibrated against gravimetric solutions prepared from CRM 112a U metal, Ames Laboratory high purity Th metal, and blank-checked ~2.5 M HNO_3 + trace HF. The gravimetric solutions, tracer stock and tracer working solution are all contained in Nalgene FEP bottles that were rigorously cleaned before use with ultrapure acids for a minimum of 3 months. Spike-sample equilibration was achieved through drying the dissolved samples down, re-dissolution in 15 M HNO_3, and refluxing at ca. 100 °C for 24 hr. Samples were then dried down and repeatedly oxidized in 15 M HNO_3 + 30% H_2O_2. After oxidation the samples were dissolved in 1 M HCl, approximately 5 mg Fe as FeCl was added, and U and Th pre-concentrated by Fe co-precipitation with ammonia. The FeCl solution used for co-precipitation was prepared from Puratronic Fe nitrate and 1 M HCl. Chemical separations of U and Th took place using Eichrom AG-1 x 8 anion resin in home-made 0.7 ml capacity ion exchange columns following procedures established by Edwards et al. (1988).

U and Th data were obtained on a Thermo Neptune Plus MC-ICP-MS fitted with an axial SEM and 9 Faraday cups. Faraday cups used for U and Th measurement were fitted with 10^{11} Ω amplifiers and the SEM was used without an RPQ to maximize SEM/Faraday gain stability. A standard Ni sample

and X skimmer cone combination was used during U analysis. The high sensitivity Jet sample and X skimmer cone combination was used for Th analysis as the samples had relatively small amounts of radiogenic ^{230}Th available for analysis. Sample delivery to the plasma was via an ESI PFA nebulizer tip (50 µl/min uptake rate) and a Cetac Aridus II desolvating nebulizer. Samples were introduced into the nebulizer in 0.2 M HCl – 0.05 M HF and the system cleaned between samples using 0.25 M HCl – 0.1 M HF following Potter et al. (2005). Instrument sensitivity was c. 500-600 V/ppm for U and c. 1100-1200 V/ppm for Th at the above sample uptake rates and with the specified cone combinations. Ar with trace N_2 was used as the transfer gas, with N_2 employed to minimize U and Th oxide production in the plasma. A standard-sample-standard bracketing protocol was employed for data collection. U was analysed in static multicollection mode with ^{234}U measured in the axial SEM and other U isotopes measured in Faraday cups. CRM 112a was used to determine the SEM/Faraday bias, and CRM 112a spiked with the IRMM 3636 ^{233}U-^{236}U tracer was used to determine the instrumental mass bias during analytical sessions (primary correction based on ^{233}U/^{236}U and checked using the spike-corrected ^{238}U/^{235}U). Th was analysed in static multicollection mode with ^{230}Th measured in the axial SEM and ^{229}Th and ^{232}Th measured on Faraday cups. Mass bias and SEM/Faraday gain was monitored during Th analytical sessions by analysing an in-house reference solution prepared from Ames Th (mainly ^{232}Th) mixed with ^{229}Th and ^{230}Th high purity tracers, previously calibrated against CRM 112a U. All raw data were corrected for hydride (~1-2 ppm) and down mass peak tailing (~2-3 ppm at 1 AMU, < 0.4 ppm at 2 AMU).

Data were reduced using an in-house spreadsheet, where ages and their uncertainties were calculated using an uncertainty propagation protocol based on McLean et al. (2011). Correction for initial Th contributions to measured activity ratios and calculation of initial [^{234}U/^{238}U] employed U series functions in Isoplot version 4 Excel 2010 add-in (see Ludwig, 2003b). Activity ratios and ages were calculated using the decay constants of (Cheng et al., 2013). A U/Th composition of [^{238}U/^{230}Th] = 0.8 ± 0.4 was used to correct all data for initial (“detrital”) Th.

Speleothem age models (Figures S1 and S2) were generated from the detritus-corrected U-Th ages (Table S1) using the StalAge algorithm (Scholz and Hoffmann, 2011). StalAge identifies and removes U-Th date outliers, followed by a Monte Carlo simulation to create the age model based upon the U-Th analyses and their associated uncertainties (see detailed discussion in Scholz and Hoffmann (2011).

Sample Name	Depth (mm)	^{238}U ppm	±2s (abs)	^{232}Th ppb	±2s (abs)	$[^{230}Th/^{232}Th]$	±2s (%)	$[^{230}Th/^{238}U]$	±2s (%)	$[^{234}U/^{238}U]$	±2s (%)	$\rho_{(08-48)}$	Age (ka) uncorr	±2s (abs)	Age (ka) corr	Age(ka) BP_{1950} corr	±2s (abs)	$[^{234}U/^{238}U]$ (initial)	±2s (abs)
**ASR**																			
**ASR 3**	**5.5**	0.07435	±0.00006	1.7266	±0.0040	3.4	±1.1	0.02002	±22	1.460	±0.30	0.0287	1.981	0.022	**1.505**	1.443	**0.338**	1.462	0.004
**ASR 9**	**7.0**	0.06272	±0.00005	0.6024	±0.0008	8.6	±2.7	0.02454	±8.1	1.357	±0.25	0.0414	2.200	0.061	**1.989**	1.927	**0.161**	1.359	0.003
**ASR 8**	**21.0**	0.1912	±0.00011	0.7761	±0.0009	35.4	±1.6	0.04618	±2.4	1.254	±0.14	0.0427	4.185	0.070	**4.089**	4.027	**0.098**	1.256	0.002
**ASR 7**	**29.0**	0.06312	±0.00007	0.1658	±0.0004	113.9	±1.8	0.09766	±1.9	1.394	±0.26	0.0033	7.951	0.150	**7.895**	7.833	**0.155**	1.403	0.004
**ASR 6**	**41.0**	0.08031	±0.00006	0.2379	±0.0015	120.4	±2.5	0.1165	±2.5	1.423	±0.18	0.0034	9.331	0.236	**9.269**	9.207	**0.239**	1.435	0.003
**ASR 5**	**62.0**	0.08491	±0.00005	0.2361	±0.0007	140.1	±1.7	0.1273	±1.8	1.486	±0.19	0.0024	9.774	0.175	**9.719**	9.657	**0.179**	1.500	0.003
**ASR 4**	**80.0**	0.07868	±0.00004	0.1380	±0.0004	226.0	±1.4	0.1299	±1.4	1.476	±0.17	0.0014	10.030	0.150	**9.995**	9.933	**0.152**	1.490	0.003
**ASR 3**	**103.0**	0.07885	±0.00008	0.1731	±0.0011	203.9	±1.8	0.1465	±1.8	1.515	±0.19	0.0011	11.073	0.208	**11.031**	10.969	**0.210**	1.532	0.003
**ASR 2**	**124.0**	0.07287	±0.00006	0.2329	±0.0006	144.0	±1.8	0.1506	±1.9	1.564	±0.21	0.0010	11.037	0.214	**10.976**	10.914	**0.218**	1.582	0.003
**ASR 1**	**152.0**	0.1501	±0.00010	0.3347	±0.0019	225.6	±2.0	0.1648	±2.0	1.515	±0.15	0.0013	12.519	0.263	**12.475**	12.413	**0.265**	1.534	0.002
**ASM**																			
**ASM 23**	**12.5**	0.09278	±0.00011	0.04940	±0.0002	10.6	±6.5	0.001712	±9.3	1.071	±0.14	0.0094	0.189	0.012	**0.175**	0.113	**0.016**	1.071	0.002
**ASM 5**	**41.5**	0.1023	±0.00010	0.05497	±0.0002	45.8	±1.3	0.007934	±1.9	1.090	±0.18	0.0079	0.811	0.011	**0.796**	0.734	**0.015**	1.090	0.002
**ASM 6**	**55.5**	0.07620	±0.00009	0.05213	±0.0003	43.2	±1.5	0.009522	±2.0	1.097	±0.22	0.0076	0.969	0.014	**0.950**	0.888	**0.020**	1.098	0.002
**ASM 7**	**69.5**	0.05702	±0.00008	0.06586	±0.0003	40.1	±1.5	0.01490	±2.1	1.094	±0.22	0.0134	1.527	0.023	**1.495**	1.433	**0.032**	1.094	0.002
**ASM 8**	**81.5**	0.06692	±0.00008	0.3414	±0.0004	12.0	±0.99	0.01871	±5.3	1.096	±0.23	0.0811	2.016	0.021	**1.877**	1.815	**0.101**	1.096	0.003
**ASM 11**	**99.5**	0.06374	±0.00008	0.07656	±0.0002	53.2	±1.2	0.02069	±1.6	1.093	±0.21	0.0149	2.116	0.025	**2.083**	2.021	**0.034**	1.094	0.002
**ASM 12**	**110.5**	0.07907	±0.00007	0.08752	±0.0002	59.9	±1.2	0.02150	±1.6	1.091	±0.19	0.0135	2.200	0.027	**2.170**	2.108	**0.035**	1.092	0.002
**ASM 16**	**138.0**	0.1079	±0.00009	0.4212	±0.0010	36.0	±0.60	0.04509	±1.7	1.175	±0.18	0.0601	4.361	0.028	**4.262**	4.200	**0.076**	1.177	0.002
**ASM 13**	**168.0**	0.05652	±0.00007	0.07282	±0.0002	114.7	±1.02	0.04822	±1.1	1.117	±0.26	0.0079	4.844	0.052	**4.809**	4.747	**0.057**	1.119	0.003
**ASM 22**	**269.5**	0.03754	±0.00007	0.3389	±0.0008	22.2	±0.99	0.06365	±2.8	1.059	±0.30	0.1231	7.011	0.076	**6.756**	6.694	**0.195**	1.060	0.003
**ASM 27**	**306.0**	0.05361	±0.00015	0.03657	±0.0002	327.3	±0.53	0.07318	±0.59	1.063	±0.13	0.0067	7.791	0.046	**7.772**	7.707	**0.048**	1.065	0.001
**ASM 28**	**320.0**	0.07523	±0.00020	0.05287	±0.0005	352.4	±0.54	0.08121	±0.56	1.061	±0.12	0.0076	8.696	0.050	**8.676**	8.614	**0.052**	1.063	0.001

Table S1: Detritus corrected U-Th ages for speleothems ASR and ASM. Data pertaining to the sampling locations and relevant U-Th measurement data from the analysis of speleothem's ASR and ASM.

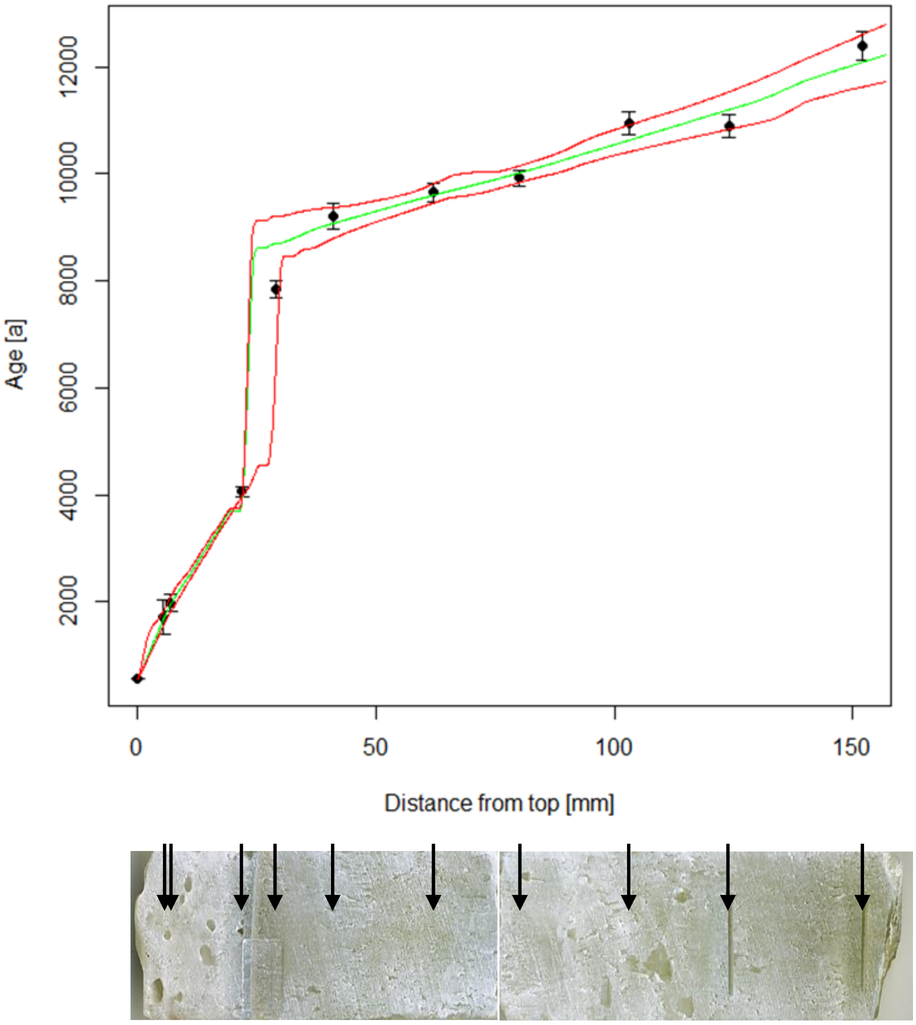

Figure S1: Speleothem ASR chronology (green line). Developed from 10 U/Th analyses on speleothem ASR using StalAge modelling; red lines denote age model error. U series dates and associated errors are presented (black diamonds) to show sample spacing. Speleothem is shown at base of figure, as are the approximate locations of U-Th analyses (black arrows).

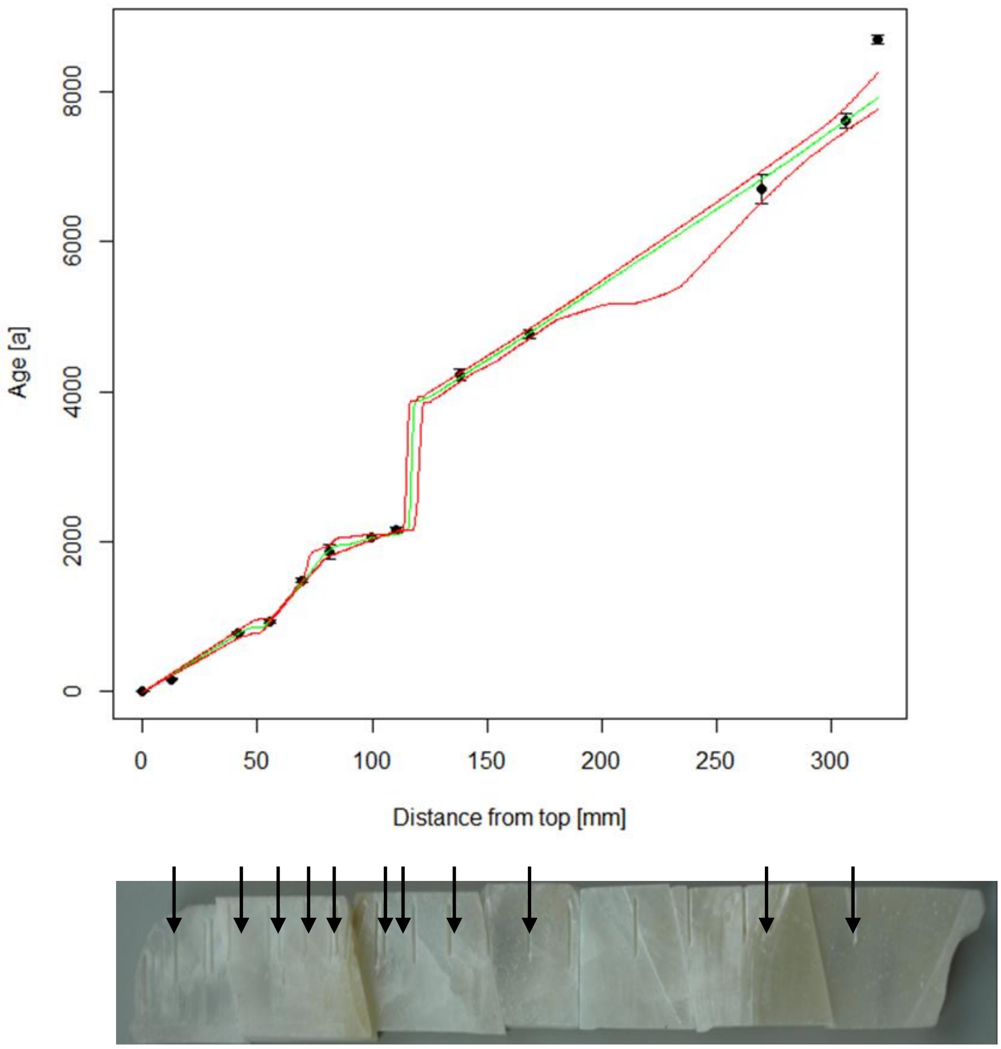

Figure S2: Speleothem ASM chronology (green line). Developed from 12 U/Th analyses on speleothem ASM using StalAge modelling; red lines denote age model error. U series dates and associated errors are presented (black diamonds) to show sample spacing. Speleothem is shown at base of figure, as are the approximate locations of U-Th analyses (black arrows).

Speleothem growth phases

Speleothem ASR grew between 12,500 and 500 BP with most rapid deposition occurring during the Younger Dryas and early Holocene (1 data point every 2-3 years). Carbonate deposition was interrupted at 8,600 ±400 BP for approximately four thousand years until ~4,000 BP. This hiatus was characterised by intermittent speleothem growth and changes to speleothem crystal structure and colour. $\delta^{18}O$ data from this period is removed from further analysis due to potential isotope fractionation during carbonate deposition. Speleothem deposition then continued during the late Holocene until 500 BP. Within this data, episodes of drying are observed at 11,800, 10,600, 9200, 8600, 6400, 4400, 2700, 1500 and 230 BP (Fig. 2).

Speleothem ASM offered a shorter record growing between 7,850 and 0 BP. Importantly this speleothem grew relatively rapidly during the mid Holocene, almost completely covering the period

of growth hiatus in ASR; a period of 650 ±300 years exists between the end of initial growth in ASR and the start of growth in ASM. One possible break in growth for ASM is identified by the StalAge algorithm between 3,800 and 2,150 BP, no obvious change in speleothem structure or colour is identified for this period. However, to ensure consistency, data related to this hiatus is also excluded from the final data set.

Stable isotope analysis

The speleothem $\delta^{18}O$ profile consists of 1244 isotope measurements from ASR and 880 from ASM, sampled using an automated micro-mill with 0.3 mm diamond encrusted drill bit along the central portion of the growth axis, creating between 50-100 µg of carbonate powder per sample. Stable isotope analysis was then undertaken at the NERC Stable Isotope Facility, British Geological Survey, using an IsoPrime isotope ratio mass spectrometer with Multiprep device; average 2σ uncertainty is 0.07‰. Isotope values are reported relative to the international VPDB standard.

Estimation of cycle periodicity and time varying amplitudes

Cycle periodicity and amplitudes have been estimated using a Dynamic Harmonic Regression (DHR) model with cycle length selection through maximisation of variance explained by single frequency DHR analysis.

Dynamic Harmonic Regression is a special case of Unobserved Components Model often used in econometrics (see e.g. Harvey and Proietti (2005)) and in this application it takes the form of:

$$y_t = T_t + C_t + I_t + e_t \qquad e_t \backsim N\{0, \sigma^2\} \qquad (1)$$

Subscript *t* denotes temporal variability of time series or its parameters and in a time series is usually the sample number. The additive components of the model, which are not observed individually, form in this case a plausible decomposition of the series in the frequency domain, and thus in Equation 1 T_t is a low frequency trend component, I_t is what is usually termed irregular component, including non-cyclic elements, e_t is the observation disturbance normally assumed to be a Gaussian distributed serially uncorrelated series, and C_t is the harmonic cyclical component.

$$C_t = \sum_{i=1}^{R} \{a_{i,t} \cos(\omega_i t) + b_{i,t} \sin(\omega_i t)\} \qquad (2)$$

In Equation 2 R is the number of frequencies $\omega_i, i = 1, \ldots, R$ used in the model, $a_{i,t}$ and $b_{i,t}, i = 1, \ldots, R$ are stochastic time varying parameters (TVP's). Estimation of these time-varying parameters is fully described in Young et al., (1999) and implemented in the CAPTAIN Toolbox for Matlab™. Implementation of a multi-frequency model is through simple addition of cyclic components, and is numerically robust due to the orthogonality of the harmonic components as long as there is a sufficient spectral separation between them, and when they are present in the data. Such multi-frequency DHR has been used in a different application in Tych et al., (2002).

One of the main stages of solving this class of problems is selection of the covariance parameters of the Kalman Filter and Fixed Interval Smoother. Their definitions and some approaches are provided and reviewed in Young et al., (1999) and Harvey and Proietti (2005). In the present context these parameters determine the time scales of variation of the estimated trend levels and amplitudes of the

cyclic components. These need to be selected in such a way that the trend component does not cover the spectrum of the cycles (that would lead to multiple colinearity in the model and associated numerical and interpretation problems.) The cyclic components' amplitudes need to be allowed to vary but to make any interpretation feasible their variability needs to be slower than the cycle they define.

It has to be noted here that the aim of tuning the Unobserved Component Models is not to achieve a perfect fit to the data. While this is achievable through choosing the variance parameters, the estimated components would then lose their interpretation, for instance the trend becoming fast varying and following the data closely. The aim in this case is to achieve objective and interpretable estimates of trend and of cyclic components, while keeping the fit to the data at a 'sensible' level.

The other parameters of the model include the cycle periodicities. These too need to be estimated objectively based on the available data. In the present analysis both periodicities are estimated using the following data based optimisation process. The set of $\omega_i, i = 1, \dots, R$ in a single cycle analysis consists of harmonics of the hypothetical frequency $\omega_0 = \frac{2\pi}{T}$, so that $\omega_i = i\omega_0$. This leads to the method applied in our analysis of finding the 'best' periodicity as the one that produces the highest power of the cyclic component signal. We use a simple quadratic norm of the cycle component:

$$\omega_0^* = \arg\max ||C_t(\omega_0)||^2 \qquad (3)$$

where $C_t(\omega_0)$ is the cyclic component (2) with the proviso that the model fit expressed as R^2 does not drop significantly. $R^2 = 1 - \frac{var(y_t - \hat{y_t})}{var(y_t)}$ is a commonly used proportion of the variance of observations y_t explained by the DHR model output $\hat{y_t}$.

In other words, we seek the cycle frequency ω_0 which gives the best proportion of the data explained by the cyclic component of the DHR model with fixed variance parameters. Such frequency can be identified by a simple univariate search using objective function (3) within Matlab ™ environment. Because of the orthogonal nature of the harmonic functions such searches can be performed individually, given their sufficient spectral separation, as orthogonality excludes interaction between the components.

For the presented data the periodicity search generates two well-defined maxima of Equation (3) at 1290 and 1490 years. These values were then used in a dual frequency DHR cycle analysis.

There is a strong indication of two separate periodicities present in the signal, as shown in Fig.S3 where the periodic component norm as in Equation 3 above (effectively: variance contribution of the periodic component) shows strong and well defined peaks at both periodicities, with a well defined trough in between them. The spectral resolution of this type of analysis is robust thanks to the orthogonality of the harmonic functional base of the DHR method, so we are confident in this result.

In terms of sensitivity of the DHR estimates to the NVR (variance parameters) the observed relationships between the periodicities of the two cycles persist throughout a broad range of the variance parameters spanning several orders of magnitude, so this dual-peak result is highly unlikely to be an artefact. The full, dual frequency DHR model explains 73.8% of the data variance (Fig. S4), showing that it is not only providing a detection tool but also indicating that the two cyclic components dominate the data. The remaining series of model residuals does not show any significant structure at longer time-scales, indicating that the DHR model is effective in explaining the entire structure of the data at the time scale longer than 150 years (10 samples).

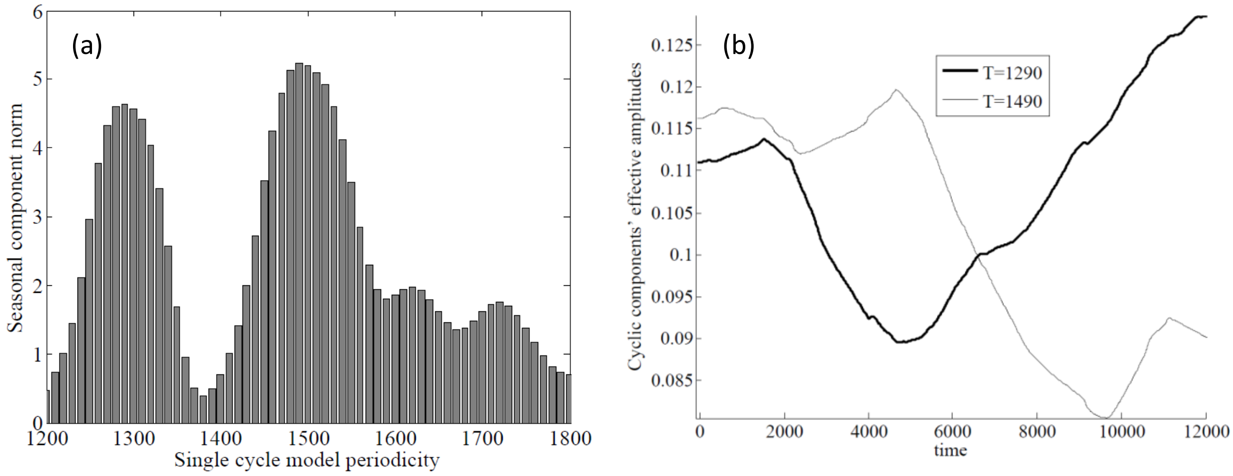

Figure S3: Dynamic Harmonic Regression frequency analysis of the combined Cueva de Asiul speleothem records. (a) Identification of dominant frequencies within the Cueva de Asiul speleothem $\delta^{18}O$ data set by Dynamic Harmonic Regression analysis. Frequencies of 1290 and 1490 years have the highest seasonal component norm. (b) The relative dominance (effective amplitude) of these cycles through the Holocene, showing a change from a dominant shorter cycle length (1290) to the longer (1490) at approximately 6500 BP.

Further hypotheses can be made on the basis of this important result including:

(a) possible change of the dominant periodicity between the two peaks, with the slower periodicity becoming dominant in the latter part of the record;
(b) possibility of the two peaks being the result of amplitude modulation of a cycle of 1.38My being modulated by a slower cycle of 19.2My

Evaluation of these hypotheses would require additional data records not available at this time.

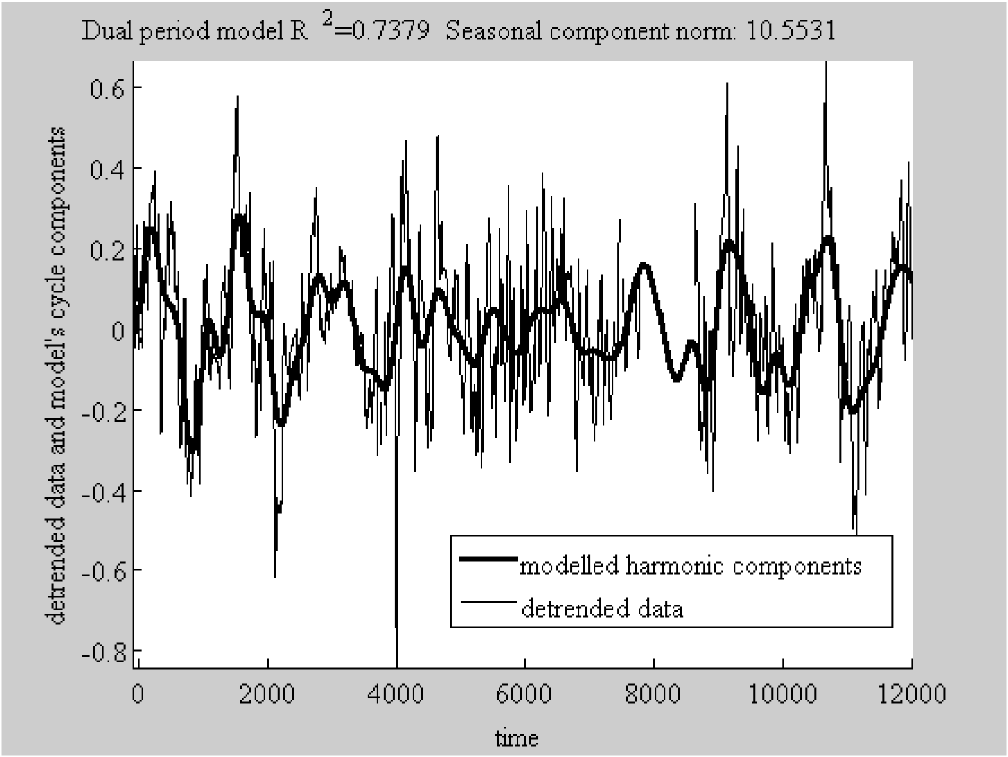

Figure S4: Combined and detrended speleothem data with the modelled harmonic components (thick black line).

Both Matlab ™ scripts used in this analysis and the CAPTAIN Toolbox for Matlab™ are available upon request from one the contributing authors (Tych), who is also one of the principal developers of the CAPTAIN Toolbox.

Record comparisons

Data pre-processing for comparisons of our data set with other climate records involved putting the compared series into a common time-base, which was regularly sampled to allow the use of time series analysis methods, including cross-correlation.

Nudge procedure: The records have been transformed from the original sampling time base into regular 15-year sampled time series. In this nudge procedure the series was first interpolated at the required 15-year grid (dt=15), then the interpolated samples which did not have a 'real' data point within dt/2 from the interpolated sample time were replaced by missing value flags (NaN in Matlab and other systems), so that the procedure did not create artefacts in the form of 'data' where there had been none originally.

Subsampling procedure: The uniformly sampled series could, if this was required, be subsampled (decimated every k-samples) to the required lower resolution by first applying an anti-aliasing low-pass no-lag filter (IRW: see e.g. Young et al, 1999, Young 2011) followed by taking every k-th sample of the smoothed (filtered) series. This procedure was used to bring the compared time series to the same temporal resolution and to remove higher frequency components in the frequency range above that of interest (smoothing). This allowed the comparison of like with like in terms of the temporal patterns of the series.

Detrending: Trend is understood here as a slow and smooth change in the series covering the lowest part of the spectrum, below the periodicities or patterns of interest (IRW trend: see e.g. Young et al, 1999, Young 2011). This amounts to low-pass filtering of the series with no static or dynamic lags involved.

Standardisation: The detrended series are then simply scaled to have standard deviation of one. This is a standard procedure in time series and generally data analysis, used when different time series need to be presented 'scale-free' to compare their patterns, including cycles.

Cross-correlation analysis: This standard time series analysis method requires the time series to be provided on the same time-base. In the classical definition, standard correlation between the samples of the time series is calculated for lags 0 (simultaneous), +-1, +-2, … so providing the level of linear relationship between the series shifted by 0, +-1 etc. (Box et al 2011).

Uncertainty estimate of Asiul record: We did not assume any initial error, the vertical uncertainty band in any comparison at reduced resolution is a conservative estimate of the 95% confidence interval resulting from smoothing the combined and detrended Asiul record required for the resolution change with no aliasing. The horizontal uncertainty is derived from the StalAge model outputs based on the individual speleothems U/Th chronologies.

The cross-analysis of series and timing errors included:

1. Comparative analysis and visualisation of both Cueva de Asiul records with timing errors. Analysing the consistency of the two Cueva de Asiul records (ASM, ASR) was based on the overlap period (550-2100AD) where both records are available (Fig S5); both Cueva de Asiul ASR and ASM series were 'nudged' into the 15-year uniform sampling and decimated with anti-aliasing; cross-correlation function was estimated with a peak close to zero lag (co-incidental) of 0.2, which was significant. This correlation was calculated for the estimated values. Bearing in mind the timing uncertainty of both of these values (Fig S5 shows the overlap period with timing uncertainty bands) the two series show similarity, justifying the combining of these records as has been done in the main analysis.

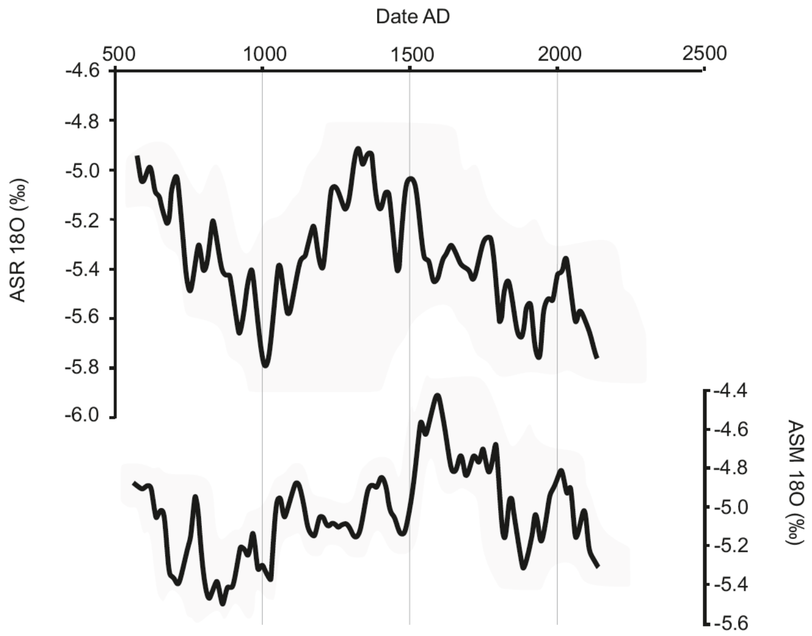

Figure S5: Combined and detrended speleothem data shown during the growth overlap period. Speleothem time series (thick black line) with U/Th error envelopes (grey bands) between 550 and 2100AD, 0 lag correlation of 0.2 is statistically significant. Visual analysis between the records indicates that a stronger relationship maybe derived if the lower resolution ASR record was allowed to vary within its U/Th error envelope.

2. Comparative analysis of Bond et al., (2001) and Cueva de Asiul records. Bond et al., data was nudged and resampled into a 60 year sampling period to allow direct comparisons with the Cueva de Asiul data subsampled with anti-aliasing into 60 year samples, then standardised as above. During the first 4000 years of the records the correlation between the two series reaches 0.505 with standard error of 0.1250, and is therefore significant. At a 15 year subsampling this relationship is 0.482 with standard error 0.129. This is visible in Fig. 3 of the main text, which shows the Bond record alongside the Cueva de Asiul data set with U/Th error plotted. If the series from present up to the hiatus in Cueva de Asiul data at 7500 is taken, then the correlation is weaker, reaching 0.165 with standard error of 0.09, so not significant. For the Early Holocene, there is no significant correlation when using the same statistical constraints as above. This is to be expected given the high timing errors at this age. Repositioning of the oldest section of the Cueva de Asiul data set (9000 – 12000BP, t= -390 years; within the boundaries of the StalAge errors) shows a high level of significant co-variation between the data sets (0.560, s.e = 0.106; Fig. S6), indicating statistically the visual similarities between the records (Fig. 3 main text) once temporal error is taken into consideration.

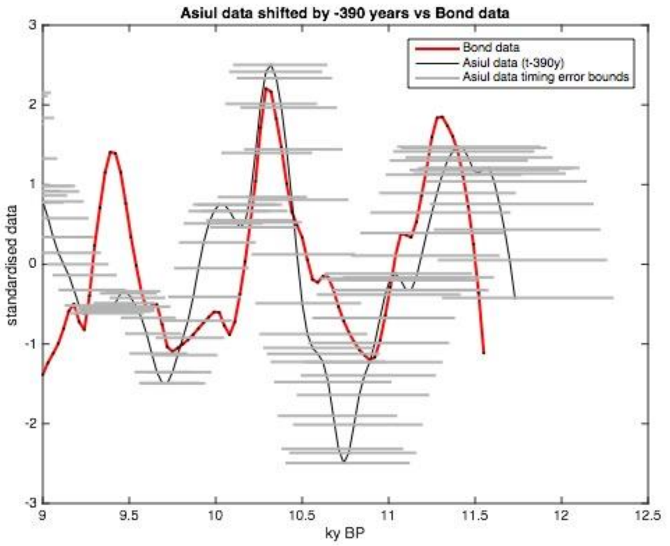

Figure S6: Normalised speleothem time series between 9000 and 12000BP vs. Bond et al., (2001) IRD record. Speleothem data is shifted by t=-390 years to maximise the correlation possible within the chronological error (from StalAge) of the speleothem time series. Strong correlations between the data set are apparent for this early Holocene section under these parameters (0.560, s.e = 0.106)

3. <u>Comparative analysis of Thornalley et al., (2009) and Cueva de Asiul records</u>
   Thornalley et al., (2009) data was nudged and resampled into 60 year sampling period, which was an average time between the samples near the beginning of the record (most recent); isolated gaps resulting from the nudging process were interpolated over using cubic splines, there were no larger gaps, where this process could introduce artefacts. The same process of detrending with variance intervention over the Cueva de Asiul hiatus period was applied as for the combined Cueva de Asiul record. This was due to the apparent step change in the Thornalley et al., (2009) data within the period of the Cueva de Asiul hiatus. The data was then standardised as above and a cross-correlation function was calculated for the two series for the entire period up to the Cueva de Asiul hiatus (0-7800BP). Correlation analysis after this period was not undertaken due to different processes acting on the ocean density record in the early Holocene (Thornalley et al., 2009). For the standardised Cueva de Asiul record the cross correlation peaked at a lag of -4 (Thornalley ocean series leading) with a cross-correlation coefficient of 0.264 (s.e. 0.089). It is also apparent that there is a strong higher frequency component in the Cueva de Asiul data, not showing in the Thornalley series. To compare like with like, the Cueva de Asiul data was smoothed to cover a similar spectrum as that of Thornalley et al., (2009) data. For this pair of series the cross-correlation function peaks at lag of about -5 (Thornalley ocean data leading) with a highly significant cross-correlation coefficient of 0.344 (s.e. of 0.090), showing a lagged relationship (Fig S7).

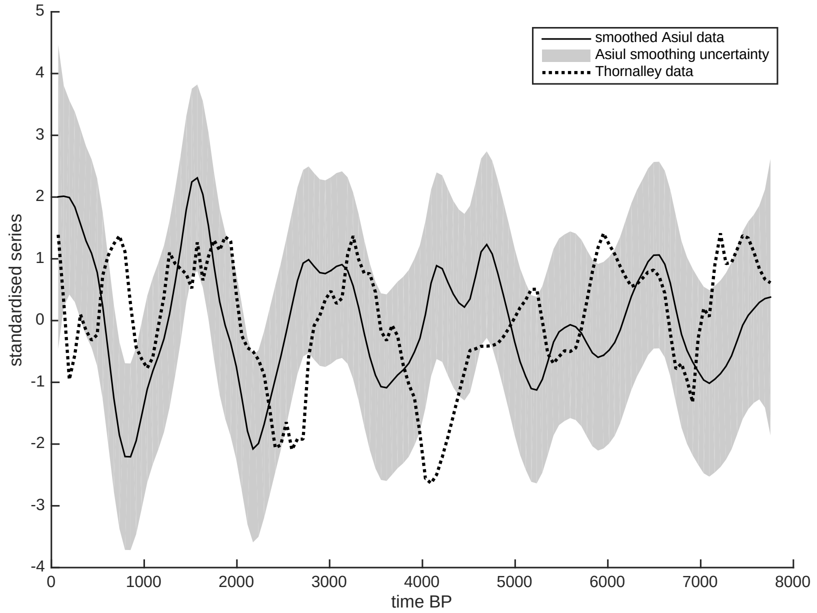

Figure S7: Comparison between ocean and atmospheric data sets. Smoothed and detrended upper ocean density stratification proxy data from core RAPiD-12-1K (dashed line, Thornalley et al., 2009) alongside smoothed and detrended Cueva de Asiul speleothem data (thin black line) showing error envelopes for the speleothem data set (grey boundaries).

4. <u>Comparative analysis of the Cueva de Asiul record with the Olsen et al., (2012) NAO record</u>: The estimated NAO record of Olsen et al., (2012) was compared with the Cueva de Asiul record (Fig. S8). The NAO estimate was detrended and standardised as described above to allow for both visualisation and application of standard time series approach to compare the patterns between the Cueva de Asiul series and the NAO series. The cross correlation function for the Cueva de Asiul and Olsen et al., (2012) series has a -60 year lag (within dating error) relationship of 0.22 which is significant, with a standard error of 0.0857.

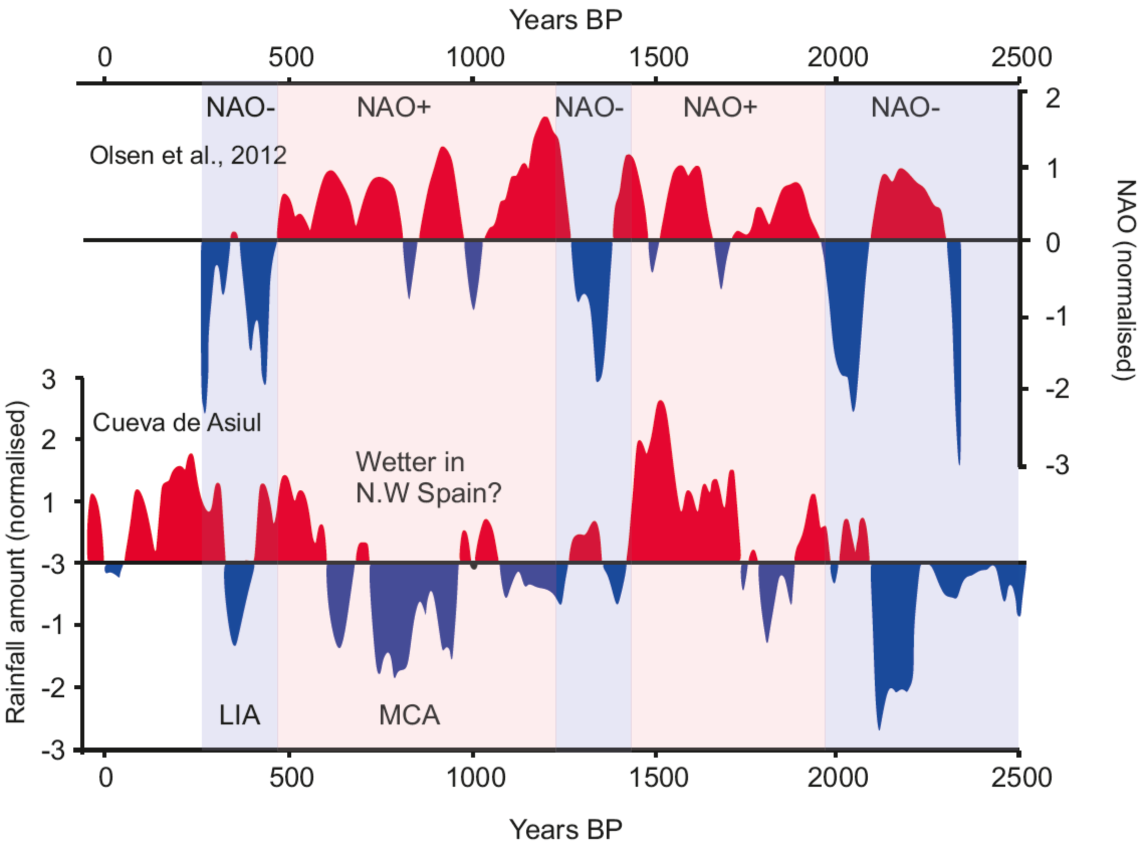

Figure S8: NAO archive of Olsen et al., (2012) with the rainfall amount record of Cueva de Asiul. Blue represents NAO- in all cases and red NAO+. Broad consistencies are observed throughout the records as shown by the cross-correlation analysis. However, the MCA which is thought to be dominated by NAO+ conditions (Trouet et al., 2009) is seen as having higher rainfall amounts (NAO-) in the Cueva de Asiul record and several other archives from north western Spain (Moreno et al., 2012 and refs. within).

Supplementary Information References

Box, G.E.P, Jenkins, G.M. and Reinsel G.C. Time series analysis, forecasting and control (fourth edition), Wiley (2013).

Cheng, H. et al. The half-lives of uranium-234 and thorium-230. *Chemical Geology*, 169, 17-33 (2000).

Edwards, R.L., Chen, J.H., and Wasserburg, G.J. ^{238}U, ^{234}U, ^{230}Th, ^{232}Th systematics and the precise measurement of time over the past 500,000 years. *Earth and Planetary Science Letters*, 81, 175-192 (1987).

Harvey, A. C. and Proietti, T, [eds.] *Readings in Unobserved Components Models*, Oxford University Press (2005).

Hendy, C.H. The isotopic geochemistry of speleothems - 1. The calculation of the effects of different modes of formation on the isotopic composition of speleothems and their applicability as palaeoclimatic indicators. *Geochimica et Cosmochimica Acta*, 35, 801-824 (1971).

Ludwig, K. R. Isoplot 3.00; a geochronological toolkit for Microsoft Excel. *Berkeley Geochronological Centre Special Publications*, 4 (2003).

McLean, N.M., Bowring, J.F. and Bowring, S.A. An algorithm for U-Pb isotope dilution data reduction and uncertainty propagation. Geochemistry, Geophysics, Geosystems, 12(6), DOI: 10.1029/2010GC003478 (2011).

Moreno, A. et al. The Medieval Climate Anomaly in the Iberian Peninsula reconstructed from marine and lake records. Quat. Sci. Rev. 43, 16–32 (2012).

Potter, E. K. et al. High precision Faraday collector MC-ICPMS thorium isotope ratio determination. *International Journal of Mass Spectrometry*, 247, 10-17 (2005).

Scholz, D. and Hoffmann, D. L. StalAge - An algorithm designed for construction of speleothem age models. *Quaternary Geochronology*, 6, 369-382 (2011).

Taylor, C. J.et al. Environmental time series analysis and forecasting with the Captain toolbox. *Environmental Modelling & Software*, 22(6), 797-814 (2007).

Thornalley, D. J. R., Elderfield, H. & McCave, I. N. Holocene oscillations in temperature and salinity of the surface subpolar North Atlantic. *Nature* **457**, 711–714 (2009).

Tremaine, D.M., Froelich, P.N and Wang, Y. Speleothem calcite farmed *in situ*: Modern calibration of $\delta^{18}O$ and $\delta^{13}C$ palaeoclimate proxies in a continuously-monitored natural cave system. *Geochimica et Cosmochimica Acta*, 75, 4929-4950 (2011).

Trouet, V. et., al. Persistent Positive North Atlantic Oscillation Mode Dominated the Medieval Climate Anomaly. Science, 324, 78–80 (2009).

Tych, W. et al. An unobserved component model for multi-rate forecasting of telephone call demand: the design of a forecasting support system. *International Journal of forecasting*, 18(4), 673-69 (2002).

Young, P. C., Pedregal, D. J. and Tych, W. Dynamic harmonic regression. *J. Forecas*t., 18: 369–394 (1999).

Young, P.C., 2011, Recursive estimation and time series analysis, Springer.
